# The Possible Role of Natural Idiotopes in Immune Memory

**DOI:** 10.1080/17402520400001645

**Published:** 2004

**Authors:** Ljiljana Dimitrijević, Snezana Živanevič-Simonović, Marijana Stojanović, Aleksandra Inić-Kanada, Irena Živković

**Affiliations:** 1 Institute of Immunology and Virology Torlak Vojvode Stepe Belgrade Serbia and Montenegro; 2 Faculty of Medicine Department of Pathophysiology Belgrade Serbia and Montenegro

## Abstract

In this paper we report on the generation of Abs possessing specificities similar to those of Abs used in immunization, and on the generation of Id and anti-Id specificities in the sera of mice immunized with commensal bacterial antigens.

The human monoclonal antibody IgM DJ (VH3/VL2) expresses natural antibody properties, natural idiotope (Y7), and specificity towards Lactic acid bacteria (LAB). When used in immunization it generates LAB-specific antibodies. Immunization with LAB, as detected in the presence of biotin-labelled mouse monoclonal anti-idiotopic antibodies Y7 and IgM DJ generates Abs1 and Abs2, respectively. These findings may imply that the recognition of bacterial motifs accords with the rules of idiotypic network theory. This theory, first proposed by Jerne in 1974 and often overlooked since, has been subject to change during the course of immunological research. Recent experiments concerning the recognition of bacterial motifs and natural memory in the immune system have inspired us in our attempt at explaining the possible role of natural Id in immune memory.


*Clinical & Developmental Immunology*, September/December 2004, VOL. 11 (3/4), pp. 281–285

### The Possible Role of Natural Idiotopes in Immune Memory

LJILJANA DIMITRIJEVIĆ[a,]*, SNEZANA ŽIVANČEVIĆ-SIMONOVIĆ[b], MARIJANA STOJANOVIĆ[a], ALEKSANDRA INIĆ-KANADA[a] and IRENA ŽIVKOVIĆ[a]

[a]*Institute of Immunology and Virology Torlak, Vojvode Stepe 458, 11152 Belgrade;* [b]*Faculty of Medicine, Department of Pathophysiology, 34000 Kragujevac, Belgrade, Serbia and Montenegro*

In this paper we report on the generation of Abs possessing specificities similar to those of Abs used in immunization, and on the generation of Id and anti-Id specificities in the sera of mice immunized with commensal bacterial antigens.

The human monoclonal antibody IgM DJ (VH3/VL2) expresses natural antibody properties, natural idiotope (Y7), and specificity towards *Lactic acid bacteria* (LAB). When used in immunization it generates LAB-specific antibodies. Immunization with LAB, as detected in the presence of biotin-labelled mouse monoclonal anti-idiotopic antibodies Y7 and IgM DJ generates Abs1 and Abs2, respectively. These findings may imply that the recognition of bacterial motifs accords with the rules of idiotypic network theory. This theory, first proposed by Jerne in 1974 and often overlooked since, has been subject to change during the course of immunological research. Recent experiments concerning the recognition of bacterial motifs and natural memory in the immune system have inspired us in our attempt at explaining the possible role of natural Id in immune memory.

*Keywords*: Anti-Id; Idiotope; Natural memory; Network

### INTRODUCTION

Jerne postulated the idiotype network theory of immune regulation some thirty years ago (Jerne, 1974). The theory has often been examined, since, but never confirmed. With an understanding of the significance of natural antibodies (NAbs), researchers were inspired to propose a model of immune regulation based on fluctuating NAbs levels. This became known as second generation immune network theory (Varela and Coutinho, 1991; Coutinho, 1995). But this model also has its weaknesses: the rejection of monoclonality, and the operation of the network in a non-activated state only. As specificity is the main characteristic of each biological function and as specific recognition is the fundamental function of antibodies (James and Tawfik, 2001), to exclude the latter's clonal nature is equivalent to excluding the entire system. A further weakness stems from the huge variation in system components with no obvious check-point markers present. And now the new idea of "epitope promiscuous receptor recognition" seems to overturn this model once and for all (Robey *et al.*, 2002a). Versatile specificities and affinities exist in immunized sera, thus the marker check-points (i.e. the clonal markers of antibody specificity and the Id markers on NAbs) necessary to monitor system function should be looked for in the substrate of steady-state non-immunized sera and on NAbs sustained by normal physiological immunoregulation. Immunization-induced Ab specificities are not always subsidiary phenomena with a temporarily protective (or harmful) role, but may be connected with immune pathology (Shoenfeld, 1994; Blank *et al.*, 2002). Another of the model's Achilles' heels was the proposition that the network functions only in a steady-state. This statement arose from the fact that no Id/anti-Id receptor-specific activation is possible in the immune system. This has raised the question of why the network is so important, whether its existence (based on just the two elements Ab1 and Ab2) is predicated upon perfect equilibrium and, subsequently, which mitogenic receptors are involved. IgM innate Abs are polyvalent and polyreactive and their main function, from the point of view of the organism's survival, is the fast recognition of pathogenic invaders (Boes *et al.*, 1998; Ochsenbein *et al.*, 1999; Kohler *et al.*, 2003). These molecules also have the characteristics necessary for cell activation (Dimitrijevic *et al.*, 1991; Robey *et al.*, 2002b).

The important role of NAbs Ids in the immune system has not been satisfactorily explained experimentally, despite the fact that their significance has been appreciated at an intuitive level for a long time (Landsteiner, 1945; Grabar, 1983; Pereira *et al.*, 1989; Avrameas, 1991). On the one hand, human serum in steady-state contains gram quantities of Ig which exhibit a myriad of perplexing

*Corresponding author. Tel.: +381-11-3975-501. Fax: +381-11-467-465. E-mail: ljiljad@eunet.yu

ISSN 1740-2522 print/ISSN 1740-2530 online © 2004 Taylor & Francis Ltd
DOI: 10.1080/17402520400001645

diversities with no satisfactory explanation of their underlying functions. Conversely, the value of experimental assays to detect specific Abs developed post-infection, or arising from autoimmunity or immunization, has been overstated and the meaning of certain specificities with regard to pathogenicity remains elusive. The spurs of various Ab specificities found in the sera of patients with autoimmune disease are of scant help: the term "pathogenic autoantibody" is fashionable but fashion, as we know, is fickle (Masllorens, 2000). More groundedly, the actual profile of serum Abs results from a process of active homeostatic selection (Poletaev and Osipenko, 2003).

In support of this last statement, we have presented the results we obtained after immunization with lactic acid bacteria (LAB) as the commensal bacterial antigen and IgM DJ, which expresses a natural Id which has been shown to possess natural antibody binding properties and which binds to LAB (Dimitrijević *et al.*, 1999). IgM DJ and mouse monoclonal anti-idiotopic antibody Y7 (MAb Y7), specific for the idiotope expressed on IgM DJ, enabled us to track the levels of immunization-induced Abs1 and Abs2 during LAB immunization. According to our results, immunization with IgM DJ generates LAB binding antibodies while LAB immunization generates high levels (redundancy) of LAB-specific Abs (Abs1) as well as Abs specific for anti-LAB Abs (Abs2). The fact that Abs2 were generated in parallel with Abs1 during LAB immunization indicates that immune response against commensally bacterial antigens is dependent on the polyclonal activation of a humoral immune response and that it is idiotypically regulated.

### MATERIALS AND METHODS

### Antigens Used for Immunization

Two antigens were used for immunization:

(1) a mixture of LAB (*B. bifidum*, *L. acidophilus*, *L. plantarum*), and
(2) IgM DJ, an antibody isolated by means of euglobulin precipitation from the serum of patient D.J. suffering from Waldenstrom macroglobulinaemia, followed by gel-exclusion chromatography on Superose-6 column (FPLC system, Pharmacia).

### Immunization Schedule

Eight-week old BALB/c female mice was immunized with IgM DJ or LAB. Antigens were applied subcutaneously, to the paw and the base of the tail, at one-week intervals in CFA, IFA and PBS. LAB was used at a concentration of $1 \times 10^9$ cells/dose, while IgM DJ was used in two concentrations, viz. 1 mg/dose and 10 μg/dose. Sera from ten BALB/c mice were collected one week after the final dose, pooled, complement-depleted and used in ELISA assay. Sera obtained form normal mice, and from normal mice injected with adjuvant only, were treated as described above and used as a control.

### Indirect ELISA for the Detection of LAB-specific Abs

Antibacterial ELISA was performed as previously described (Dimitrijević *et al.*, 1999). Briefly, LAB was adhered on poly-L-lysine (PLL) pre-coated plates (centrifugation at 500 *g* followed by 1 h incubation) and covalently coupled to PLL by the addition of 50 μl 0.5% glutardialdehyde (15 min) to stabilize adherence. Plates were saturated with 0.5% BSA/0.1 M glycine/PBS for 2 h at 37°C. Antibody binding was detected by the use of biotin-labelled (-B) anti-mouse IgG and streptavidin–peroxidase conjugate. Enzyme reaction was developed for 30 min with *o*-phenylen-diamine as substrate and then stopped by the addition of 2M $H_2SO_4$. Optical density (OD) was read at 492/620 nm (Multiscan Ascent Labsystem).

### Competitive ELISA for Detection of Abs1 and Abs2

For inhibition ELISA, two antibodies were used: human monoclonal Ab IgM DJ and its specific anti-idiotypic antibody MAb Y7 IgG1 isotype, isolated over the IgM DJ CL 4B immunoaffinity column (Dimitrijević *et al.*, 1992). Abs were biotin-labelled as described previously (Dimitrijević *et al.*, 1999). Both MAb Y7 and IgM DJ were labelled highly specific, as the concentrations necessary for 50% inhibition of binding or saturation were equal in each case. The binding of 100 ng of either biotin-labelled IgM DJ or MAb Y7 to its relevant component adsorbed to plastic (Y7 MAb or IgM DJ, respectively) were detected after pre-incubation (1 h at room temperature) with different dilutions of LAB-immunized sera.

### RESULTS

### Detection of LAB-specific Antibodies

LAB-specific antibodies were generated in mice immunized with either LAB or LAB-specific Ab, IgM DJ (Fig. 1). In LAB-immunized mice, these Abs could be assigned as Abs1, while in IgM DJ immunized mice anti-LAB Abs could be regarded as part of the Abs2 population. Comparing the groups of mice immunized with IgM DJ, a positive correlation between the IgM DJ concentration used and the level of induced anti-LAB Abs was observed. It was of interest that higher levels of LAB-specific antibodies were achieved in the sera of mice immunized with a high dose of IgM DJ than in the LAB-immunized mice (Fig. 1). Levels of anti-LAB antibodies were also detected in the sera of control mice.

### Detection of Ab1 and Ab2 in LAB-immunized Sera

Abs1 and Abs2 were detected in the sera of LAB-immunized mice by competitive ELISA (Fig. 2). Abs1 were detected by measuring the inhibition of MAb Y7-B binding to immobilized IgM DJ in the presence of various sera dilutions. Complete inhibition was achieved at a sera dilution of 1:25 (Fig. 2A). Inhibition of IgM DJ-B binding to MAb Y7 in the presence of sera from LAB-immunized mice was used for Ab2 determination, when sera diluted 1:200 completely inhibited MAb Y7–IgM DJ-B interaction (Fig. 2B).

### DISCUSSION

In this paper we have reported, firstly, on the generation of antibodies possessing specificity similar to that of the Ab expressing natural idiotope which was used for immunization, and, secondly, on the simultaneous induction of Id and anti-Id specificities, Abs1 and Abs2, in the sera of mice immunized with LAB. It is the case with our method that the immunization of mice with anti-DNA (or Id) Abs also results in the production of anti-DNA Abs. It has already been shown that anti-DNA antibodies can express complementarities similar to those existing in DNA molecules (Cottet *et al.*, 1994; Cottet and Bordenave, 1994) and can be induced by antibodies of any specificity, not necessarily those that are DNA-specific. Polyreactive, repetitive Ig fragments, which bear linked B and T cell epitopes can, when presented to appropriate B cells, stimulate the cell, become endocytosed and processed in the same way as normal antigens for MHC class II presentation to T cells (Fehr *et al.*, 1997). In this way "natural" Abs specific for DNA can be formed in a normal human or animal (Kalsi *et al.*, 1996). Diversification of this

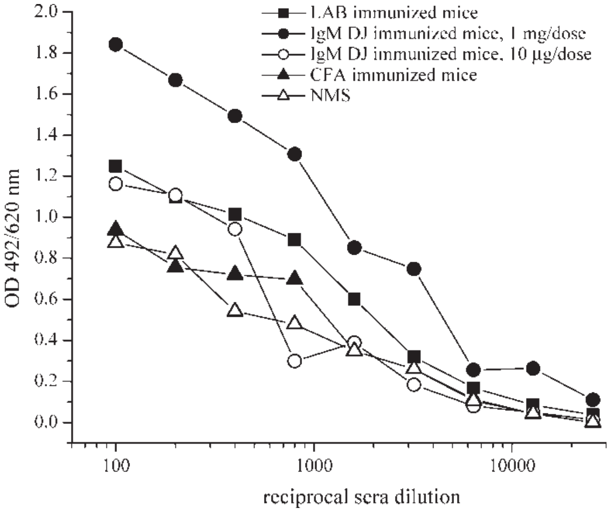

FIGURE 1 Binding of sera, obtained from mice immunized with LAB and with differing concentrations of IgM DJ (1 mg and 10 μg), to LAB coated microplates. Normal mouse sera, and sera from mice administered adjuvant only, were used as controls. Each point represents a mean of triplicate values of ten pooled sera.

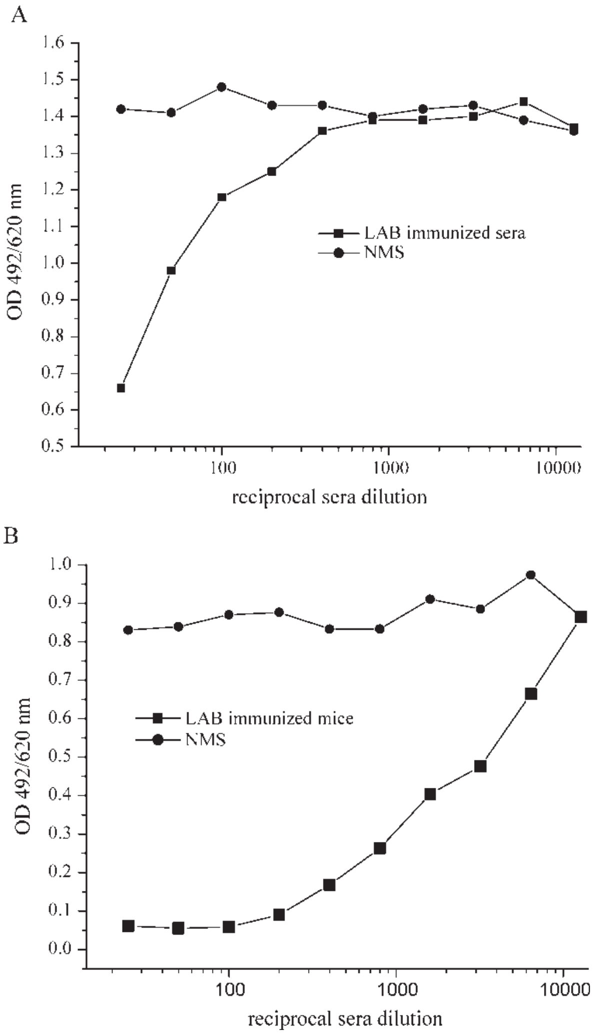

FIGURE 2 The analysis of LAB-immunized mice sera detected an idiotopic complementary structure. (A) Competitive inhibition of binding of idiotope (biotin-labelled IgM DJ 100 ng/ml) to its relevant anti-idiotopic antibody (Y7 mouse MAb) coated onto microplate in the presence of lactobacillus immunized mouse serum. (B) Competitive inhibition of binding of anti-idiotopic antibody (biotin-labelled Y7 100 ng/ml) to its relevant idiotope (IgM DJ) coated onto microplate in the presence of lactobacillus immunized mouse serum. BALB/c immunization was done with three doses ($10^9$ bacterial cells/dose) applied subcutaneously at weekly intervals in CFA, IFA and PBS. Sera were collected seven days after the final dose. Each point represents a mean of triplicate values of ten pooled sera.

response, which upsets the balance of the steady-state levels of non-specific T cell stimulation, generates immune pathology, epitope spread and unrecognized Ags in antibody-dependent immune pathologies. In our systematology, the existence of repetitive protein determinants sustained in Abs1 and Abs2 could explain the generation of the same specificity as sustained in the Ag used for immunization. The conserved protein structures, natural idiotopes, are the substrate for natural immune memory and tolerance. In general, similar interspecies and cross-reactive Ids indicate that such structures may function, at an evolutionary level, as the chatelaines of fortresses besieged by sophisticated and versatile micro-organisms (Janeway, 2001; Marchalonis *et al.*, 2002).

The production of identical antibody (Ab) specificities upon immunization (Reilly and Root, 1986; Hebert *et al.*, 1990) with either the antigen (Ag) or its specific idiotope (Id) (Forni *et al.*, 1980; Stein and Soderstorm, 1984; Hanson *et al.*, 2003) expressed on monoclonal antibody (MAb) with natural Ab characteristics, supports the conceptual framework proposed by Jerne (Jerne, 1974), which may be expressed in a biological sense by the regulation of the immune response. In other words, immunization with NAb preferentially stimulates direct expression of VH germline genes, without further diversification of the Id response (Schiff *et al.*, 1986). This may well generate a certain degree of redundancy. Idiotopes as well as NAbs gain the qualifier *recurrent* (Lundkvist *et al.*, 1987) when their redundancy and regulatory role (Araujo *et al.*, 1987) in a network become obvious. If we accept that the recurrent idiotope on natural Ig has a regulatory role in memory or tolerance induction, and if we label it as Abs1, then the question, which arises is: what are the characteristics of its complementary Abs2? Although Abs2 interact with Abs1 through some of their CDR regions, Abs2 have also to partially duplicate the biological structure and function of Abs1 in order to ensure the biological functionality of Ab1s. The most important biological function of Abs1 is the provision of immediate protection against invaders: for steady-state functionality in this protective capacity, Abs2 should represent a pool of monoclonal components with differing specificities and affinities providing basal levels of Abs1. In the case of memory induction, Abs2 should have very specific characteristics both at the structural level and in terms of concentrations. MHC peptide presentation of V regions of Abs2 should provide the stimulation or depression of Abs1. When the presented peptides originate from Abs2 (probably light chains) CDRs acquire structural similarities with Abs1 H CDR3 (Alfonso *et al.*, 2002), the levels of Abs1 rising as a consequence of non-specific T receptor stimulation, which for their part stimulate the B cells with similarly presented peptides, irrespective of origin.

This type of response is designed to amplify the B cell Ig response to bacterial challenge, through the repetitive protein structure which, by analogy to Pathogen Associated Molecular Pattern (PAMP) (Janeway and Medzhitov, 2002), could be designated as Self Associated Molecular Pattern (SAMP). Microbial carbohydrates and lypopolysaccharides express regularly-spaced repeating epitopes that induce multivalent membrane Ig (mIg) cross-linking on the B cell surface. This mediates potent mIg-dependent B cell signalling which by itself induces only B cell proliferation, but which, in concert with cytokines and/or polyclonal microbial activators, can co-stimulate Ig secretion and Ig class switching. Dangers arise when microbial interaction with B cells induces receptor-mediated signalling [through Toll receptor stimulation (Vasselon and Detmers, 2002)] thereby activating the immune system, an interaction which, by virtue of idiotypic cross-reactivity, cannot be dealt with by the natural Abs (i.e. the polyreactive V regions and T cell receptors sustained in natural idiotope) which are steady-state players in the system.

Ig V regions and TCR SAMP sequences, in a way similar to the PAMP, could use the same receptors (Tolls are, after all, pathogen recognition receptors) to interact with and provide the basal levels of stimulation which exist in steady-state. SAMP sequences, in competition with PAMP, could provide protection against evolutionarily conserved microbial structures as part of an evolutionarily conserved natural memory. If such competition does not exist, as in the case of LAB (which may be regarded as a tolerogen due to its redundancy and its presence early in maturation) immunization, natural idiotopes are stimulated with no further diversification of the humoral immune response, lending support to Jerne's concept of the role of idiotypes in immune regulation.

### *Acknowledgements*

We thank Dušanka Nikolić (The Torlak Institute of Immunology and Virology, Belgrade) for kindly providing the LAB preparation.
This investigation was supported by a 1239 grant from the Ministry of Science and Technology of the Republic of Serbia.

### *References*

Alfonso, M., Díaz, A., Hernández, A.M., *et al.* (2002) "An anti-idiotype vaccine elicits a specific response to *N*-glycolyl sialic acid residues of glycoconjugates in melanoma patients", *J. Immunol.* **168**, 2523–2529.

Araujo, P.M., Holmberg, D., Martinez, A.C. and Coutinho, A. (1987) "Idiotypic multireactivity of 'natural' antibodies. 'Natural' anti-idiotypes also inhibit helper cells with cross-reactive clonotypes", *Scand. J. Immunol.* **25**, 497–505.

Avrameas, S. (1991) "Natural autoantibodies: From "horror autotoxicus" to "gnothi seauton"", *Immunol. Today* **12**, 154–159.

Blank, M., Krause, I., Fridkin, M., *et al.* (2002) "Bacterial induction of autoantibodies to beta2-glycoprotein-I accounts for the infectious etiology of antiphospholipid syndrome", *J. Clin. Investig.* **109**, 797–804.

Boes, M., Prodeus, A.P., Schmidt, T., Carrol, M.C. and Chen, J. (1998) "A critical role of natural immunoglobulin M in immediate defense against systemic bacterial infection", *J. Exp. Med.* **188**, 2381–2386.

Cottet, M.H. and Bordenave, G. (1994) "Epitope complementarity and idiotypic interactions: a study of idiotypic-like interactions between anti-cytidine and anti-guanosine A/J mouse monoclonal antibodies-II. Analysis of the gene repertoire used to encode the variable regions of these antibodies", *Mol. Immunol.* **31**, 75–76.

Cottet, M.H., Denoyelle, C. and Bordenave, G. (1994) "Epitope complementarity and idiotypic interactions: a study of idiotypic-like interactions between anti-cytidine and anti-guanosine A/J mouse monoclonal antibodies-I. Characterization of these interactions", *Mol. Immunol.* **31**, 65–74.

Coutinho, A. (1995) "The network theory—21 years later", *Scand. J. Immunol.* **42**, 3–8.

Dimitrijevic, L., Živančević-Simonović, S., Odrljin, T., Radulović, M., Jovanović, D. and Isaković, K. (1991) "T cell proliferation induced by murine monoclonal anti-idiotypic antibody", In: Imhof, A.B., Berrih-Aknin, S. and Ezine, S., eds, Lymphatic Tissues and *In Vivo* Immune Responses (Marcel Dekker, New York), Vol. 37, pp 221–225.

Dimitrijević, L., Radulović, M., Ćirić, B., Odrljin, T., Jankov, R. and Marzari, R. (1992) "Immunochemical characterization of a murine monoclonal anti-idiotypic antibody", *J. Immunoassay* **13**, 181–196.

Dimitrijević, L., Radulović, M., Ćirić, B., *et al.* (1999) "Human monoclonal IgM DJ binds to ssDNA and human commensal bacteria", *Hum. Antibodies* **9**, 37–45.

Fehr, T., Bachmann, F.M., Bucher, E., *et al.* (1997) "Role of repetitive antigen paterns for induction of antibodies against antibodies", *J. Exp. Med.* **185**, 1785–1792.

Forni, L., Coutinho, A., Kohler, G. and Jerne, N.K. (1980) "Antibodies induce the production of antibodies of the same specificity", *PNAS* **77**, 1125–1128.

Grabar, P. (1983) "Autoantibodies and physiological role of immunoglobulins", *Immunol. Today* **4**, 337–340.

Hanson, L.A., Korotkova, M., Lundin, S., *et al.* (2003) "The transfer of immunity from mother to child", *Ann. N Y Acad. Sci.* **987**, 199–206.

Hebert, J., Bernier, D., Boutin, Y., Jobin, M. and Mourad, W. (1990) "Generation of anti-idiotypic and anti-anti-idiotypic monoclonal antibodies in the same fusion. Support of Jerne's network theory", *J. Immunol.* **144**, 4256–4261.

James, L.C. and Tawfik, D.S. (2001) "Catalytic and binding polyreactivities shared by two unrelated proteins: The potential role of promiscuity in enzyme evolution", *Protein Sci.* **10**, 2600–2607.

Janeway, A.C., Jr (2001) "How the immune system works to protect the host from infection. A personal view", *PNAS* **98**, 7461–7468.

Janeway, J.C., Jr and Medzhitov, R. (2002) "Innate immune regulation", *Annu. Rev. Immunol.* **20**, 197–216.

Jerne, N.K. (1974) "Towards a network theory of the immune system", *Ann. Immunol.* **125C**, 373–382, (Paris).

Kalsi, J.K., Martin, A.C., Hirabayashi, Y., *et al.* (1996) "Functional and modeling studies of the binding of human monoclonal anti-DNA antibodies", *Mol. Immunol.* **33**, 471–483.

Kohler, H., Bayry, J., Nicoletti, A. and Kaveri, V.S. (2003) "Natural autoantibodies as tools to predict the outcome of immune response?", *Scand. J. Immunol.* **58**, 285–298.

Landsteiner, K. (1945) The Specificity of Serological Reactions, 2nd ed. (Harvard University Press, Cambridge, MA).

Lundkvist, I., Ivars, F., Holmberg, D. and Coutinho, A. (1987) "The immune response to bacterial dextrans V. A "dominant" idiotype in IgCHb mice", *J. Immunol.* **138**, 4395–4401.

Marchalonis, J.J., Kaveri, S., Lacroix-Desmazes, S. and Kazatchkine, D.M. (2002) "Natural recognition repertoire and evolutionary emergence of the combinatorial immune system", *FASEB J.* **16**, 842–848.

Masllorens, O.A. (2000) "Autoimmune disease and physiological autoimmunity: recognition of self evolution of the concept of autoimmunity", *Allergol. Immunol. Clin.* **15**, 5–12.

Ochsenbein, F.A., Fehr, T., Lutz, C., *et al.* (1999) "Control of early viral and bacterial distribution by natural antibodies", *Science* **286**, 2156–2159.

Pereira, P., Bandeira, A., Coutihno, A., Marcos, M.A., Toribio, M. and Martinez, A.C. (1989) "V region connectivity in T cell repertoires", *Ann. Rev. Immunol.* **7**, 209–249.

Poletaev, A. and Osipenko, L. (2003) "General network of natural autoantibodies as immunological homunculus (Immunculus)", *Autoimmun. Rev.* **2**, 264–271.

Reilly, T.M. and Root, R.T. (1986) "Production of idiotypic and anti-idiotypic antibodies by BALB/c mice in response to immunizations with glucagon, vasopressin, or insulin: supporting evidence for the network concept", *J. Immunol.* **137**, 597–602.

Robey, I.F., Edmundson, A.B., Schluter, S.F., Yocum, D.E. and Marchalonis, J.J. (2002a) "Specificity mapping of human anti-T cell receptor monoclonal natural antibodies: defining the properties of epitope recognition promiscuity", *FASEB J.* **16**, 642–652.

Robey, F.I., Schluter, F., Akporiaye, E., Yocum, E.D. and Marchalonis, J.J. (2002b) "Human monoclonal natural autoantibodies against the T-cell receptor inhibit interleukin-2 production in murine T cells", *Immunology* **105**, 419–429.

Schiff, C., Milili, M., Hue, I., Rudikoff, S. and Fougereau, M. (1986) "Genetic basis for expression of the idiotypic network. One unique Ig VH germline gene accounts for the major family of Ab1 and Ab3 (Ab1$'$) antibodies of the GAT system", *J. Exp. Med.* **163**, 573–587.

Shoenfeld, Y. (1994) "Idiotypic induction of autoimmunity: a new aspect of the idiotypic network", *FASEB J.* **8**, 1296–1301.

Stein, K.E. and Soderstorm, T. (1984) "Neonatal administration of idiotype or antiidiotype primes for protection against *Escherichia coli* K13 infection in mice", *J. Exp. Med.* **160**, 1001–1011.

Varela, J.F. and Coutinho, A. (1991) "Second generation immune networks", *Immunol Today* **12**, 159–166.

Vasselon, T. and Detmers, A.P. (2002) "Toll receptors: a central element in innate immune responses", *Infect. Immun.* **70**, 1033–1041.

